# IDH-Mutant Diffuse Glioma: From Metabolic Origins to Targeted Therapy

**DOI:** 10.3390/jcm15166387

**Published:** 2026-08-18

**Authors:** Tadeja Urbanic-Purkart

**Affiliations:** 1 Department of Neurology, Medical University of Graz, Auenbruggerplatz 22, 8036 Graz, Austria; tadeja.urbanic-purkart@medunigraz.at; 2 Division of Neuroradiology, Vascular and Interventional Radiology, Medical University of Graz, Auenbruggerplatz 22, 8036 Graz, Austria

**Keywords:** IDH-mutant diffuse glioma, D-2-hydroxyglutarate, glioma-associated epilepsy, vorasidenib, precision neuro-oncology

## Abstract

**Background/Objectives:** Isocitrate Dehydrogenase (IDH)1/2-mutant diffuse gliomas represent a biologically distinct subgroup of adult brain tumors in which eary metabolic reprogramming and accumulation of the oncometabolite D-2-hydroxyglutarate (D-2HG) drive epigenetic, immunologic, and clinical characteristics, including a high burden of glioma-associated epilepsy. This review summarizes the molecular and metabolic consequences of IDH mutations, their role in glioma-associated epilepsy, and the evolving impact of mutant IDH-targeted therapies in contemporary neuro-oncology. **Methods:** We conducted a narrative review of key molecular, translational, imaging, and clinical studies on IDH-mutant diffuse gliomas. The literature included the 2021 (World Health Organization) WHO Classification of Tumours of the Central Nervous System, studies investigating D-2HG biology and glioma-associated epilepsy, and prospective clinical trials and real-world evidence evaluating IDH-targeted therapies and contemporary antiseizure management. Particular emphasis was placed on vorasidenib, advanced metabolic imaging, and emerging liquid biopsy approaches. **Results:** IDH mutations are early driver events that promote D-2HG accumulation, resulting in widespread epigenetic reprogramming, metabolic dysregulation, and an immunosuppressive tumor microenvironment. D-2HG has also been implicated in the development of glioma-associated epilepsy, although the underlying mechanisms remain incompletely understood. Advances in integrated histomolecular diagnostics, magnetic resonance spectroscopy, amino acid positron emission tomography, and cerebrospinal fluid liquid biopsy have improved disease classification and treatment monitoring. Mutant IDH inhibitors, particularly vorasidenib, prolong progression-free survival, delay the need for subsequent treatment, and reduce intratumoral D-2HG concentrations, and have shown encouraging early signals of improved seizure control and preserved health-related quality of life in patients with grade 2 IDH-mutant gliomas, although this evidence remains preliminary and requires confirmation in larger prospective studies. **Conclusions:** IDH-mutant diffuse gliomas exemplify precision neuro-oncology, in which a single metabolic alteration informs diagnosis, disease monitoring, and targeted therapeutic approach. Additionally, ongoing studies are expected to further define the role of IDH inhibition across different disease stages and in combination with immunotherapy and standard treatments. Lastly, future clinical trials should systematically incorporate seizure outcomes, neurocognitive function, patient-reported outcomes, and immunologic endpoints to optimize both tumor control and quality of life.

## 1. Introduction

Diffuse gliomas are the most common primary malignant tumors of the adult central nervous system and remain associated with substantial neurological morbidity and cancer-related mortality despite advances in surgery, radiotherapy, chemotherapy, and molecular diagnostics. Approximately one-quarter of diffuse gliomas harbor mutations in the IDH1 or IDH2 genes. Although WHO grade 2 diffuse gliomas generally have a more favorable prognosis than higher-grade tumors, they remain infiltrative neoplasms with a persistent risk of recurrence and malignant progression during long-term follow-up [1,2].

Until the introduction of molecular diagnostics, diffuse lower-grade gliomas were classified predominantly according to histopathological features, despite substantial biological heterogeneity and variable clinical behavior. The discovery of recurrent IDH1 and IDH2 mutations fundamentally changed this paradigm by establishing IDH-mutant diffuse gliomas as a biologically and clinically distinct entity [3,4]. This conceptual shift reshaped the WHO classification of diffuse gliomas and provided the biological rationale for the development of mutation-specific, metabolically targeted therapeutic strategies, which form the focus of this review [1].

This narrative review is based on a literature search of PubMed/MEDLINE, Embase, and ClinicalTrials.gov, completed on 1 August 2026, using combinations of the terms IDH mutation, isocitrate dehydrogenase, D-2-hydroxyglutarate, diffuse glioma, glioma-associated epilepsy, vorasidenib, mutant IDH inhibitor, and precision neuro-oncology. Given the narrative rather than systematic design of this review, articles and clinical trial records were selected based on relevance, methodological quality, and contribution to the current understanding of IDH-mutant glioma biology, diagnostics, and treatment, supplemented by hand-searching of reference lists and recent neuro-oncology conference proceedings.

While most IDH-mutant diffuse gliomas occur sporadically, rare germline susceptibility variants, including rs55705857 at chromosome 8q24, and other inherited loci are associated with an increased risk of developing IDH-mutant astrocytomas and oligodendrogliomas [5,6].

The discovery of recurrent IDH1 and IDH2 mutations has fundamentally transformed the classification and biological understanding of diffuse gliomas. The IDH1 and IDH2 genes are located on chromosomes 2q33.3 and 15q26.1, respectively, and mutations have also been identified in several other solid and hematologic malignancies, including acute myeloid leukemia, myelodysplastic syndrome, intrahepatic cholangiocarcinoma, and chondrosarcoma [3,7,8].

**Figure 1:**
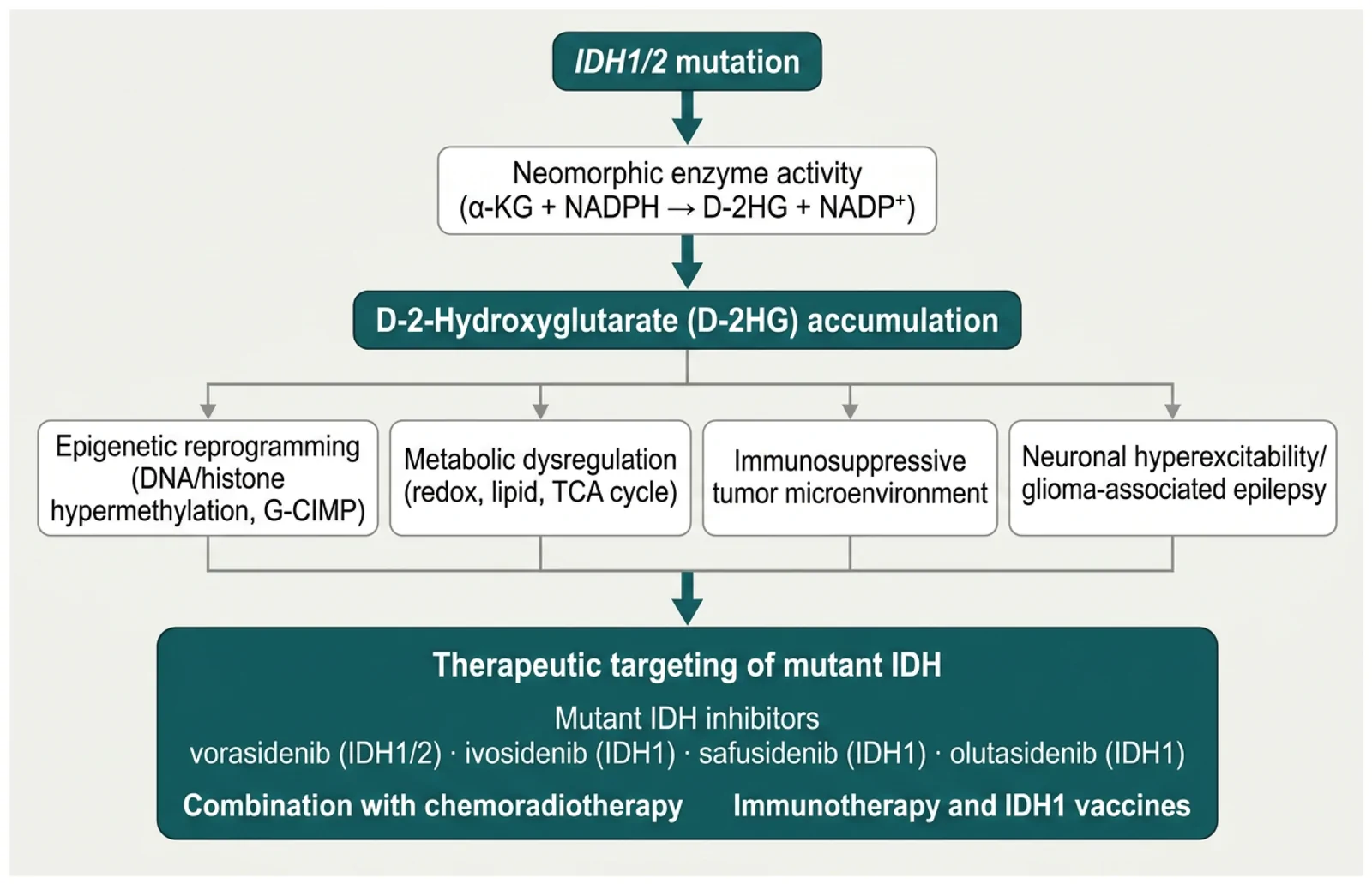
Schematic overview of the biological cascade in IDH-mutant diffuse glioma, from the initiating IDH1/2 mutation and D-2-hydroxyglutarate (D-2HG) accumulation to its downstream epigenetic, metabolic, immunologic, and epileptogenic consequences, and current therapeutic strategies targeting mutant IDH. Abbreviations: NADP^+^: oxidized nicotinamide adenine dinucleotide phosphate, NADPH: nicotinamide adenine dinucleotide phosphate, G-CIMP: glioma CpG island methylator phenotype, TCA cycle: tricarboxylic acid cycle, IDH1/2: Isocitrate Dehydrogenase 1 and 2.

The isocitrate dehydrogenase family comprises three isoforms: IDH1, which is localized in the cytoplasm and peroxisomes, and the mitochondrial enzymes IDH2 and IDH3. Unlike IDH1 and IDH2, IDH3 has not been established as a recurrent oncogenic driver in human cancers. Nonetheless, these enzymes play essential roles in cellular metabolism, including oxidative phosphorylation, glutamine metabolism, lipogenesis, glucose sensing, and maintenance of cellular redox homeostasis [3]. Mutations in IDH1 and IDH2 confer a neomorphic enzymatic activity that converts α-ketoglutarate (α-KG) into the oncometabolite D-2-hydroxyglutarate (D-2HG) [9], and the resulting accumulation of D-2HG inhibits α-KG-dependent dioxygenases, leading to widespread DNA and histone hypermethylation, impaired cellular differentiation, and establishment of the glioma CpG island methylator phenotype (G-CIMP) (Figure 1) [10,11,12].

Because IDH mutations occur early during gliomagenesis and are maintained throughout tumor evolution, they serve as robust diagnostic biomarkers and attractive therapeutic targets [3,7,13]. Their clinical importance is reflected in the 2021 World Health Organization (WHO) Classification of Tumours of the Central Nervous System, in which IDH mutation status constitutes a key diagnostic criterion. At the same time, advances in molecular diagnostics, including proton magnetic resonance spectroscopy (^1^H-MRS), DNA methylation profiling, and liquid biopsy approaches, have improved the non-invasive diagnosis and monitoring of patients with IDH-mutant gliomas; however, these techniques are not yet widely implemented in routine clinical practice [14].

The therapeutic relevance of targeting IDH mutations was established by the phase III INDIGO trial, in which vorasidenib significantly prolonged progression-free survival and delayed the need for subsequent therapeutic intervention in patients with residual or recurrent WHO grade 2 IDH-mutant gliomas [15,16]. Subsequent perioperative studies and emerging real-world evidence have demonstrated that mutant IDH inhibition reduces intratumoral D-2HG concentrations and is associated with improved disease control [17,18]. This review summarizes the molecular, metabolic, and clinical consequences of IDH mutations in diffuse gliomas and discusses how these advances are shaping contemporary approaches to diagnosis, disease monitoring, and targeted therapy.

## 2. IDH Mutation as the Earliest Tumorigenic Event in Diffuse Gliomas

The discovery of recurrent IDH1 and IDH2 mutations in diffuse gliomas established metabolic dysregulation as an initiating event of gliomagenesis rather than merely a consequence of malignant transformation [3,13,19]. In adults, IDH1 and IDH2 mutations occur almost exclusively and are present in the majority (>80%) of WHO grade 2 and grade 3 diffuse gliomas and grade 4 IDH-mutant astrocytomas (formerly known as secondary glioblastomas); however, they are comparatively uncommon in pediatric gliomas [2,3,7,19]. Additionally, their remarkable stability across spatially distinct tumor regions and throughout disease progression identifies them as founder, or trunk, mutations that precede most other recurrent genomic alterations [7,13].

The biochemical hallmark of IDH-mutant gliomas is the neomorphic production of D-2-hydroxyglutarate (D-2HG) from α-ketoglutarate (α-KG) [9]. Under physiological conditions, IDH1 and IDH2 catalyze the reversible oxidative decarboxylation of isocitrate to α-KG while generating NADPH, with mutant IDH enzymes fundamentally altering this reaction. The canonical IDH1 R132H mutation confers gain-of-function enzymatic activity in which the catalytically active heterodimer, composed of one wild-type monomer and one mutant IDH1 monomer, reduces α-KG to D-2HG in an NADPH-dependent reaction. In contrast, homodimers consisting of two mutant IDH1 monomers lack catalytic activity [9,12]. Since IDH mutations arise early during gliomagenesis and are clonally conserved, they are detectable across tumor evolution and recurrence and may already be present in oligodendrocyte progenitor-like cells [7,13,20,21].

Low-frequency IDH1-mutant cells have also been identified in brain tissue beyond the histological tumor margin, suggesting that the initiating mutation extends into the infiltrative zone surrounding the radiologically visible lesion. This finding may contribute to the diffuse growth pattern of these tumors and helps explain why complete surgical resection is rarely achievable [21].

Following the initiating IDH mutation, diffuse gliomas diverge into two principal molecular pathways. Astrocytomas typically acquire TP53 mutations and ATRX loss, while oligodendrogliomas are characterized by combined 1p/19q codeletion and TERT promoter mutations [22,23]. Accordingly, the 2021 WHO Classification of Tumours of the Central Nervous System recognizes three principal categories of adult diffuse gliomas:

- Astrocytoma, mutant IDH;
- Oligodendroglioma, mutant IDH and codeleted 1p/19q;
- Glioblastoma, wild-type IDH.

This integrated histomolecular classification has substantially improved diagnostic accuracy, prognostic stratification, and therapeutic decision-making and represents a cornerstone of precision neuro-oncology. IDH-mutant astrocytoma is the most common subtype of diffuse glioma and typically presents in adults during the third or fourth decade of life. Most tumors are diagnosed as CNS WHO grade 2 or grade 3, while grade 4 tumors are less common. Patients with grade 2 or grade 3 disease generally have a more favorable prognosis, with median overall survival approaching 10 years, compared with approximately 3–7 years for grade 4 IDH-mutant astrocytoma [1,15,24].

In this context, it is important to distinguish glioblastoma, IDH-wildtype, CNS WHO grade 4, from astrocytoma, IDH-mutant, CNS WHO grade 4, the latter corresponding to tumors historically referred to as secondary glioblastoma. Under the current WHO classification, the designation glioblastoma is reserved exclusively for IDH-wildtype diffuse astrocytic tumors with grade 4 histological or molecular features, whereas progressive IDH-mutant tumors are classified as astrocytoma, IDH-mutant, CNS WHO grade 4, irrespective of whether they arise de novo or evolve from a lower-grade precursor [1].

This distinction is also reflected in population-based data. An analysis of the German Cancer Registry (2009–2021) found that WHO grade 4 tumors accounted for approximately 68% of newly diagnosed diffuse gliomas, whereas grade 2 and grade 3 tumors each accounted for approximately 9% [8]. However, the majority of grade 4 tumors are glioblastoma, IDH-wildtype: population-based data from the United States (CBTRUS, 2018–2022) report an age-adjusted incidence of 2.51 per 100,000 for grade 4 IDH-wildtype astrocytoma/glioblastoma, compared with only 0.08 per 100,000 for astrocytoma, IDH-mutant, CNS WHO grade 4—an approximately 31-fold difference in incidence, corresponding to IDH-mutant astrocytoma accounting for only a small minority (approximately 2.3%) of grade 4 diffuse astrocytic tumors [25]. Together, these data underscore that although grade 4 tumors constitute the majority of adult diffuse gliomas, an astrocytoma, IDH-mutant, CNS WHO grade 4, remains comparatively rare relative to glioblastoma, IDH-wildtype.

### Molecular Drivers of Malignant Progression

Although the IDH mutation is the initiating event in gliomagenesis, progression toward higher-grade disease requires the acquisition of additional molecular and biological alterations superimposed on the characteristic IDH-mutant, TP53-mutant, ATRX-deficient background of astrocytomas. Recent integrative genomic and epigenomic studies of matched initial and recurrent tumor samples indicate that malignant transformation of IDH-mutant astrocytoma is driven by three interrelated biological processes: enhanced cellular proliferation, loss of mature glial identity (dedifferentiation), and remodeling of the extracellular matrix [26,27]. These transcriptional and cell-state changes occur alongside the acquisition of high-risk genetic alterations, including cyclin-dependent kinase inhibitor 2A/2B (CDKN2A/B) homozygous deletion, cyclin-dependent kinase 4 (CDK4) and platelet-derived growth factor receptor alpha (PDGFRA) amplification, loss of retinoblastoma 1 (RB1) loss, and phosphatidylinositol-4,5-bisphosphate 3-kinase catalytic subunit alpha (PIK3CA) or phosphoinositide-3-kinase regulatory subunit 1 (PIK3R1) mutations, together with progressive genome-wide DNA hypomethylation, indicating that malignant progression reflects both genetic and epigenetic reprogramming [26,28].

These findings are further supported by cIMPACT-NOW Update 12, which proposes refinement of pathology-based grading of IDH-mutant astrocytoma through incorporation of molecular biomarkers. Specifically, PDGFRA amplification is considered consistent with CNS WHO grade 4 behavior and is proposed as a grade 4 criterion, while CDKN2A/B homozygous deletion is already an established CNS WHO grade 4 criterion. PIK3CA/PIK3R1 mutations, EGFR alterations, MYCN amplification, and RB-pathway alterations—including CDK4/CDK6/CCND2 amplification, RB1 homozygous deletion or mutation, and inactivating CDKN2A mutation—are considered most consistent with CNS WHO grade 3 in the absence of grade 4 histologic or genetic features. G-CIMP-low and A (astrocytoma)_IDH_HG(high grade) methylation signatures are likewise considered consistent with at least CNS WHO grade 3, with assignment to grade 3 or 4 depending on other grade 4 criteria. These recommendations were informed by outcome-based risk stratification, with cIMPACT-NOW Update 12 considering median overall survival of <6 years as compatible with CNS WHO grade 4 behavior, <10 years as compatible with grade 3, and ≥10 years as compatible with grade 2 in IDH-mutant astrocytomas [29]. Collectively, these observations support the use of integrated molecular biomarkers, rather than histopathological features alone, for grading and risk stratification of IDH-mutant astrocytomas, and may increasingly guide surveillance strategies and eligibility for molecularly stratified clinical trials.

Among these progression-associated alterations, PDGFRA has emerged as a particularly relevant and potentially actionable biomarker. Located on chromosome 4q12, PDGFRA encodes a transmembrane receptor tyrosine kinase that regulates oligodendrocyte development and activates the RAS/MAPK and PI3K/AKT signaling pathways, thereby influencing cellular proliferation, migration, and survival. PDGFRA amplification is well described in glioblastoma, where it represents the second most frequently altered receptor tyrosine kinase after EGFR and has been associated with adverse prognosis [30]. Notably, its prevalence increases with histological grade in IDH-mutant astrocytomas, and it is associated with aggressive clinical behavior [29]. Earlier tyrosine kinase inhibitors, including imatinib, sorafenib, nilotinib, and sunitinib, showed limited clinical benefit in glioma, largely reflecting inadequate central nervous system penetration; in contrast, newer, more selective inhibitors such as avapritinib have demonstrated promising preclinical activity and favorable brain penetration in PDGFRA-altered high-grade glioma models [31]. Clinical evidence for PDGFRA-directed therapy currently remains limited largely to pediatric and young adult PDGFRA-altered high-grade gliomas; nonetheless, these findings provide proof of principle that PDGFRA represents a potentially actionable therapeutic target warranting further evaluation in molecularly selected adult patients with IDH-mutant glioma.

## 3. Metabolic Reprogramming and the D-2HG Oncometabolite

The hallmark of IDH-mutant gliomas is the accumulation of the oncometabolite D-2-hydroxyglutarate (D-2HG), which promotes gliomagenesis through competitive inhibition of α-ketoglutarate (α-KG)-dependent dioxygenases [10]. Intratumoral D-2HG concentrations can reach 20–30 mmol/L, which is several orders of magnitude higher than those observed in normal brain tissue. This inhibition reduces the activity of TET DNA demethylases and Jumonji-domain histone demethylases, resulting in widespread DNA and histone hypermethylation and establishment of the glioma CpG island methylator phenotype (G-CIMP) [11,12]. These epigenetic alterations impair cellular differentiation, promote the maintenance of a stem-like cellular state, and thereby support gliomagenesis [7,13].

The biological consequences of mutant IDH extend well beyond epigenetic dysregulation. Sustained NADPH consumption disrupts cellular redox homeostasis, while D-2HG-driven metabolic reprogramming alters amino acid metabolism, lipid metabolism, mitochondrial function, extracellular matrix remodeling, and cellular differentiation [32,33,34]. In addition, increasing evidence indicates that IDH-mutant gliomas establish an immunosuppressive tumor microenvironment. In contrast, D-2HG modulates the function of both innate and adaptive immune cells, thereby promoting immune evasion and providing a strong biological rationale for therapeutic strategies combining mutant IDH inhibitors with immunotherapy (Figure 1) [35,36].

## 4. Glioma-Associated Epilepsy as a Consequence of Metabolic Reprogramming

Seizures are among the most common presenting symptoms of IDH1/2-mutant diffuse gliomas, occurring particularly frequently in lower-grade, cortical, and non-enhancing tumors [37,38]. Contemporary series report seizure rates exceeding 70%, with a substantial impact on quality of life and functional independence. These rates are markedly higher than in IDH-wildtype glioblastoma, in which distinct metabolic alterations appear to promote epileptogenesis through different mechanisms than those operating in IDH-mutant tumors [39]. Despite advances in antiseizure therapy, seizure control remains incomplete in a considerable proportion of patients [38].

Experimental evidence suggests that D-2-hydroxyglutarate (D-2HG) contributes to glioma-associated epilepsy by altering the peritumoral neuronal microenvironment. Although D-2HG is structurally similar to glutamate, current evidence indicates that it does not directly mimic glutamate at NMDA receptors, making direct NMDA receptor activation an uncertain mechanism of epileptogenesis. Instead, D-2HG is thought to promote neuronal hyperexcitability primarily through metabolic reprogramming, including disruption of the tricarboxylic acid (TCA) cycle, induction of hypermetabolic states, dysregulation of intracellular calcium homeostasis, altered glutamatergic signaling, increased synaptic glutamate availability, and activation of mTOR-dependent pathways. Nevertheless, the precise contribution of D-2HG to epileptogenesis remains incompletely understood, and whether it directly drives seizure generation within the surrounding brain tissue continues to be investigated [37,38,40].

Current management practice focuses on optimizing antiseizure medication (ASM) therapy, with levetiracetam, lacosamide, and lamotrigine representing commonly used first-line agents. Emerging evidence suggests that perampanel, an AMPA receptor antagonist, may provide additional benefits to patients with IDH-mutant, MGMT-methylated gliomas, while radiochemotherapy can reduce seizure frequency by more than 50%, even in the absence of substantial radiographic tumor shrinkage [41,42,43]. More recently, preliminary clinical data suggest that vorasidenib may further reduce seizure burden beyond conventional treatment, consistent with its ability to suppress D-2HG production; long-term seizure stabilization has been reported, especially in patients with oligodendroglioma. Postoperative seizure risk itself may also be modifiable by IDH-directed therapy, although this potential benefit likewise requires confirmation in larger cohorts [44]. However, these observations are based on limited patient numbers and short follow-up, and larger prospective studies with longer-term outcomes are needed before firm conclusions can be drawn [16,45,46].

Collectively, these findings suggest that pharmacological inhibition of mutant IDH may improve both tumor and seizure control, highlighting seizure burden as an increasingly important therapeutic target and patient-centered clinical endpoint in future studies of IDH-mutant diffuse gliomas, although current evidence for this benefit remains preliminary [16,35,37].

## 5. Precision Diagnostics and Monitoring

The 2021 World Health Organization (WHO) Classification of Tumours of the Central Nervous System established integrated histologic and molecular diagnosis as the international standard for classifying adult diffuse gliomas [1]. In routine clinical practice, diagnostic evaluation typically begins with immunohistochemistry for IDH1 R132H, which identifies the vast majority of IDH1/2-mutant gliomas [5,47]. Tumors with compatible histologic features but negative IDH1 R132H immunostaining should undergo IDH1 and IDH2 sequencing to detect less common variants affecting IDH1 Arg132 (including R132S, R132C, R132G, and R132L) or IDH2 Arg172 (R172K) [1,24,47].

Additional molecular markers enable distinction between the principal IDH-mutant glioma subtypes. Specifically, the loss of ATRX expression together with abnormal p53 immunoreactivity supports an astrocytoma diagnosis, while whole-arm 1p/19q codeletion defines oligodendroglioma [1,24,47]. Other biomarkers, including homozygous CDKN2A/B deletion, MGMT promoter methylation, and TERT promoter status, provide important prognostic and therapeutic information and increasingly influence treatment planning and surveillance strategies. Notably, homozygous CDKN2A/B deletion is sufficient to classify an IDH-mutant astrocytoma as a CNS WHO grade 4 astrocytoma, irrespective of histologic features [1,47,48]. Additionally, genome-wide DNA methylation profiling has further improved diagnostic accuracy, particularly in tumors with an ambiguous morphology or atypical molecular features, and is increasingly used in specialized neuropathology centers as a complement to conventional histomolecular evaluation [24].

Beyond establishing the WHO diagnosis, integrated molecular diagnostics provides a biological framework that informs individualized clinical decision-making, including the extent of surgical resection, follow-up strategy, radiotherapy and chemotherapy planning, the selection of targeted therapies, and eligibility for molecularly stratified clinical trials.

### 5.1. Metabolic Imaging

One of the most distinctive features of IDH-mutant gliomas is the ability to noninvasively image their characteristic metabolic product, D-2-hydroxyglutarate (D-2HG). Proton magnetic resonance spectroscopy (^1^H-MRS) enables in vivo detection of D-2HG and provides a highly specific metabolic biomarker of IDH1/2-mutant gliomas [6,49]. Additionally, advances in spectral acquisition, spectral-editing techniques, and post-processing algorithms have substantially improved the reliability of D-2HG detection despite spectral overlap with glutamate, glutamine, and γ-aminobutyric acid resonances [6,50].

Longitudinal studies have demonstrated that serial ^1^H-MRS measurements of D-2HG correlate with tumor evolution and provide pharmacodynamic evidence of target engagement during mutant IDH inhibition. This is particularly valuable because conventional structural MRI does not always capture the earliest biological effects of treatment [49,50,51]. Nevertheless, the technical complexity of ^1^H-MRS and its limited availability outside specialized centers continue to restrict its widespread clinical implementation.

Conventional MRI remains indispensable for anatomical assessment; however, metabolic responses may precede measurable changes in tumor volume. During treatment with mutant IDH inhibitors, rapid suppression of D-2HG is often solely followed by gradual reductions in tumor size, reflecting cellular differentiation and slowed tumor growth rather than acute cytotoxicity [17,49,52,53,54]. Consequently, stable disease on conventional MRI during the first months of therapy should not necessarily be interpreted as treatment failure. Imaging responses should instead be assessed according to the Response Assessment in Neuro-Oncology (RANO) 2.0 criteria, the current standardized framework for evaluating treatment response and disease progression in adult diffuse gliomas, which integrates conventional MRI findings with clinical status and corticosteroid use [55].

[18F]Fluoroethyl-L-tyrosine positron emission tomography ([18F]FET PET) is an amino acid PET imaging technique that provides complementary metabolic information beyond conventional MRI and may further improve tumor characterization and treatment monitoring in patients with IDH1/2-mutant gliomas. Additionally, FET uptake is generally more frequent and more intense in oligodendrogliomas than in astrocytomas, and tracer uptake tends to increase with WHO grade despite the considerable overlap between grades. Limited spatial concordance between FET uptake and MRI abnormalities indicates that metabolically active tumor regions often extend beyond contrast-enhancing areas and do not fully correspond to FLAIR abnormalities [56]. In patients receiving mutant IDH inhibitors, preliminary studies suggest that serial FET PET can detect metabolic responses within 5–8 weeks, even when conventional MRI remains unchanged, highlighting its potential as an early imaging biomarker of therapeutic response [57]. However, approximately 30% of IDH1/2-mutant gliomas demonstrate little or no amino acid tracer uptake, and prospective studies are still needed to establish the predictive and prognostic value of amino acid PET [56,57].

Recent volumetric MRI analyses, together with emerging FET PET data, further support the need for a broader response-assessment framework. Tumor growth rates may decline before measurable radiographic shrinkage occurs, and maximal imaging responses can require many months of treatment. These observations support the integration of volumetric MRI and metabolic imaging biomarkers into future response-assessment strategies for patients with IDH-mutant gliomas [57,58].

### 5.2. Liquid Biopsy

Liquid biopsy offers a minimally invasive approach to molecular disease monitoring in gliomas, although its clinical utility remains limited by the low concentration of circulating analytes and the restrictive effects of the blood–brain barrier. Measuring D-2-hydroxyglutarate (D-2HG) expression via liquid chromatography–mass spectrometry in tumor tissue, cerebrospinal fluid (CSF), and plasma represents one metabolomic strategy, with CSF generally demonstrating superior analytical performance compared with peripheral blood [59,60].

CSF-derived circulating tumor DNA (ctDNA) analysis allows for the identification of IDH mutations and additional genomic alterations associated with tumor progression or therapeutic resistance, thereby supporting longitudinal molecular monitoring, particularly when conventional imaging findings are inconclusive [61,62]. Plasma-based cell-free DNA represents a more accessible, less invasive alternative and has also been associated with survival outcomes in glioma patients, although its sensitivity for IDH mutation detection remains lower than that of CSF-derived ctDNA [63]. Additionally, emerging multimodal approaches that integrate ctDNA analysis, DNA methylation profiling, extracellular vesicles, and computational classification algorithms may further improve diagnostic sensitivity and enable more personalized, minimally invasive monitoring of disease evolution. At present, liquid biopsy complements rather than replaces tissue-based histopathological diagnosis; however, it is becoming increasingly valuable in selected clinical scenarios where molecular evidence of recurrence or treatment resistance may influence the timing of therapeutic intervention.

## 6. Therapeutic Targeting of Mutant IDH

The recognition of IDH mutations as early, clonally conserved driver events has transformed a defining biological feature of diffuse gliomas into a therapeutic target. Mutant IDH inhibitors suppress the production of D-2-hydroxyglutarate (D-2HG), thereby targeting the metabolic alteration that underlies many of the downstream epigenetic, metabolic, and differentiation abnormalities characteristic of IDH-mutant gliomas. Consequently, mutant IDH inhibition represents one of the clearest examples of mechanism-based therapy derived from advances in cancer metabolism research [2,13,15,32].

### 6.1. Targeted Inhibition of Mutant IDH

Before the introduction of mutant IDH-targeted therapy, the management of WHO grade 2 IDH-mutant gliomas was based on maximal safe surgical resection followed by active surveillance in low-risk patients or postoperative radiotherapy combined with alkylating chemotherapy—most commonly procarbazine, lomustine, and vincristine (PCV) or, in selected cases, temozolomide—in accordance with EANO recommendations and phase III evidence supporting RT-PCV in high-risk low-grade gliomas [15,16,64,65,66].

The therapeutic potential of targeting mutant IDH was established by the phase III INDIGO trial, which evaluated vorasidenib, a brain-penetrant dual inhibitor of mutant IDH1 and IDH2, in patients with residual or recurrent, predominantly non-enhancing WHO grade 2 IDH1/2-mutant gliomas following surgery who did not require immediate U.S. FDA-approved chemoradiotherapy. Compared with a placebo, vorasidenib significantly prolonged median progression-free survival (27.7 vs. 11.1 months), delayed the time to next therapeutic intervention, and reduced tumor growth rates. Additionally, the treatment was generally well tolerated, with grade ≥3 transaminase elevation representing the principal clinically relevant adverse event [15].

Extended follow-up confirmed the durability of these clinical benefits, which led to the approval of vorasidenib in August 2024 for adults and adolescents (≥12 years) with resected WHO grade 2 IDH1/2-mutant gliomas, followed by European Medicines Agency (EMA) approval in 2025 [15,16]. Delaying the need for radiotherapy and chemotherapy is particularly relevant for younger patients, as it may postpone treatment-related neurocognitive and systemic toxicities while preserving long-term quality of life.

Although vorasidenib has become the preferred mutant IDH inhibitor in this setting, other agents, including ivosidenib, remain clinically relevant, particularly when vorasidenib is unavailable. Emerging real-world evidence further suggests that a predominantly non-enhancing radiographic phenotype, rather than WHO grade alone, may better predict durable disease control with IDH-directed therapy [17,54]. In parallel, several next-generation mutant IDH inhibitors are currently undergoing clinical evaluation and may further expand treatment options for patients with IDH-mutant gliomas [67].

### 6.2. Combination with Cytotoxic Chemotherapy

Combination strategies with cytotoxic chemotherapy are also under active clinical investigation. Temozolomide (TMZ) exerts its cytotoxic effect predominantly through the formation of O6-methylguanine DNA adducts; although this lesion accounts for only a minor fraction of TMZ-induced DNA damage, it is the principal driver of mismatch repair (MMR)-dependent cytotoxicity and apoptosis. Resistance to TMZ is determined largely by MGMT promoter methylation status and by the functional integrity of the MMR pathway, both of which also influence the efficacy of alkylating chemotherapy in IDH-mutant glioma [68]. Building on this mechanistic rationale, the combination of vorasidenib with TMZ is currently being evaluated in the phase Ib/II NCT06478212 trial, which enrolls patients with recurrent grade 2–4 or newly diagnosed grade 4 IDH-mutant glioma [69], as well as in the phase III ALLIANCE/A072301 trial in newly diagnosed WHO grade 3 IDH-mutant astrocytoma (Table 1). These studies represent the translation of mutant IDH inhibition from monotherapy in grade 2 disease toward combination approaches in higher-grade IDH-mutant glioma.

### 6.3. Resistance, Patient Selection, and Treatment Sequencing

As mutant IDH inhibitors move into broader clinical use, questions regarding resistance, patient selection, and treatment sequencing are gaining increasing importance. Mechanisms of resistance to mutant IDH inhibition remain incompletely characterized in glioma; by analogy with experience in IDH-mutant myeloid malignancies, plausible mechanisms include the emergence of second-site IDH mutations, activation of alternative metabolic or signaling pathways, and clonal selection of IDH-wildtype or otherwise high-risk subclones, although direct evidence in glioma is still limited. Appropriate patient selection currently relies on tumor grade, extent of residual disease, and radiographic phenotype, with emerging evidence suggesting that a predominantly non-enhancing pattern, rather than WHO grade alone, may better predict durable benefit from IDH-directed therapy [17,54]. The optimal sequencing of surgery, radiotherapy, chemotherapy, and IDH-targeted therapy remains unresolved, particularly for grade 3 and grade 4 IDH-mutant astrocytoma, and is being directly addressed by the ongoing VIGOR and ALLIANCE trials (Table 1). In parallel, multidisciplinary consensus efforts—such as a recent Delphi study among Spanish neuro-oncology experts—have proposed practical recommendations for managing grade 2 IDH-mutant glioma in the vorasidenib era, while highlighting persisting areas of uncertainty, including the clinical role of liquid biopsy and the optimal timing of IDH-inhibitor initiation [70].

### 6.4. Limitations and Practical Considerations

Several limitations should be considered when interpreting the current evidence base for mutant IDH inhibition. Clinical evidence supporting vorasidenib is currently strongest for non-enhancing, residual or recurrent WHO grade 2 IDH-mutant diffuse glioma, as established in the INDIGO trial; data in contrast-enhancing or higher-grade (WHO grade 3–4) tumors remain limited and are the subject of ongoing investigation (Table 1) [15,16]. Long-term safety data, although reassuring to date, remain relatively immature; grade ≥ 3 transaminase elevation is the most frequently reported clinically relevant adverse event, and hepatotoxicity warrants continued monitoring during extended treatment [15]. Practical considerations, including drug cost and geographic variability in access to vorasidenib and to advanced molecular and imaging diagnostics, may also affect the feasibility of implementing IDH-targeted management in routine clinical practice. Other mutant IDH inhibitors, including ivosidenib, safusidenib, and olutasidenib, together with the immunotherapeutic and combination strategies discussed below, provide alternative or complementary options, and their comparative role relative to vorasidenib continues to be defined.

## 7. Future Horizons and Ongoing Trials

The success of the INDIGO trial has established mutant IDH inhibition as a therapeutic strategy in diffuse glioma and has stimulated a broad range of studies aimed at expanding its role across different disease stages and treatment settings. Current research focuses on extending the benefits of IDH inhibition to higher-grade gliomas, integrating targeted therapy with standard treatment, and exploring rational combinations with immunotherapy [61].

### 7.1. Ongoing Clinical Trials

Several clinical trials are evaluating vorasidenib beyond the setting investigated in INDIGO (Table 1). The phase III VIGOR trial (NCT06809322) is assessing vorasidenib as maintenance therapy following standard chemoradiotherapy in patients with CNS WHO grade 2 or 3 IDH-mutant astrocytoma, whereas the phase III ALLIANCE/A072301 trial (NCT07215910) is evaluating vorasidenib in combination with adjuvant temozolomide following radiotherapy in patients with newly diagnosed CNS WHO grade 3 IDH-mutant astrocytoma. Clinical development is also extending to CNS WHO grade 4 disease: the phase Ib/II study NCT06478212 evaluated vorasidenib in combination with temozolomide in recurrent grade 2–4 IDH-mutant glioma and newly diagnosed grade 4 IDH-mutant astrocytoma and is currently active but no longer recruiting. Building on this strategy, the planned randomized, double-blind, placebo-controlled phase III S095032-301 study will evaluate vorasidenib plus temozolomide specifically in patients with grade 4 IDH1- or IDH2-mutant astrocytoma. In addition, the multinational IDHEAL4U study is investigating vorasidenib in patients with CNS WHO grade 4 IDH-mutant astrocytoma, collectively extending mutant IDH inhibition into more advanced disease settings [71,72,73].

Beyond targeted therapy alone, the immunosuppressive effects of D-2-hydroxyglutarate (D-2HG) provide a strong biological rationale for combining mutant IDH inhibition with immunotherapy. Ongoing phase I studies are evaluating vorasidenib in combination with pembrolizumab (NCT05484622), while the ViCToRy trial (NCT05609994) is evaluating it with an IDH1 peptide vaccine in patients with recurrent WHO grade 2 or 3 IDH1-mutant gliomas [74,75]. In parallel, immunotherapeutic strategies targeting mutant IDH1 continue to evolve. Long-term follow-up from the NOA-16 trial has demonstrated the safety and sustained immunogenicity of an IDH1 R132H peptide vaccine, while the AMPLIFY-NEOVAC study is evaluating peptide vaccination in combination with PD-L1 blockade [76,77]. Furthermore, frontline vaccination strategies targeting mutant IDH1 have shown promising immunogenicity in newly diagnosed astrocytoma, and recent systematic reviews support the potential synergy between IDH inhibitors and immunotherapy [78,79].

### 7.2. Advances in Drug Delivery

Beyond molecularly targeted agents, advances in drug-delivery technology may further improve the treatment of IDH-mutant glioma. Hyaluronic acid-based nanocarriers represent one such strategy, exploiting interactions with the extracellular matrix and with CD44, which is expressed by glioma cells, to enhance drug penetration across the blood–brain barrier and improve intratumoral distribution [80]. Although preclinical studies have demonstrated enhanced tumor targeting and drug delivery in glioblastoma models, these nanocarrier platforms have not yet been specifically validated in adult IDH-mutant diffuse lower-grade glioma, and their relevance to this molecular subgroup remains investigational and warrants dedicated preclinical evaluation.

The development of mutant IDH-targeted therapy also extends beyond vorasidenib. Other inhibitors, including safusidenib, are currently being evaluated in a phase III clinical trial (NCT05303519) [81], while olutasidenib is under investigation in combination with temozolomide following radiotherapy in newly diagnosed pediatric and young adult patients with WHO grade 3 or 4 IDH1-mutant gliomas (NCT06161974) [82]. Collectively, these studies are expected to further define the optimal sequencing and combination of IDH-targeted therapies and expand their role across different grades and molecular subtypes of IDH-mutant gliomas [32,79].

## 8. Conclusions

IDH1/2-mutant diffuse gliomas have become a paradigm of precision neuro-oncology, in which a defining metabolic alteration has been translated into biomarker-driven diagnosis, disease monitoring, and mechanism-based therapy. Moreover, the integration of histopathology, molecular genetics, epigenetics, and advanced metabolic imaging has substantially improved diagnostic accuracy, prognostic stratification, and treatment selection. At the same time, mutant IDH inhibitors, particularly vorasidenib, have established a new therapeutic paradigm by prolonging progression-free survival and delaying the need for chemoradiotherapy, with preliminary evidence suggesting a potential to reduce seizure burden while preserving health-related quality of life, although this benefit requires confirmation in larger, longer-term studies.

Beyond direct antitumor effects, growing insights into the biological consequences of D-2-hydroxyglutarate (D-2HG), including its roles in metabolic reprogramming, glioma-associated epilepsy, and immune modulation, continue to expand opportunities for mechanism-based therapeutic interventions. Thus, ongoing clinical trials evaluating IDH inhibition in higher-grade gliomas, in combination with chemoradiotherapy, immune checkpoint inhibitors, and IDH1-directed vaccines, are expected to further define the role of targeted therapy across different stages of disease.

Future clinical studies should integrate patient-reported outcomes, seizure control, neurocognitive function, and molecular biomarkers alongside conventional radiographic endpoints to ensure that advances in tumor-directed therapy translate into meaningful and durable clinical benefits for patients with IDH-mutant diffuse gliomas.

## Figures and Tables

**Table 1:** Selected Clinical Trials of Next-Generation IDH Inhibitors in IDH-Mutant Gliomas.

Trial	NCT/Identifier	Phase	What It Examines	Sponsor
VIGOR	NCT06809322	Phase III	Vorasidenib maintenance versus placebo after first-line chemoradiotherapy in WHO grade 2–3 IDH-mutant astrocytoma, with progression-free survival as the primary endpoint.	European Organisation for Research and Treatment of Cancer (EORTC)
ALLIANCE/A072301	NCT07215910	Phase III	Vorasidenib versus placebo in combination with adjuvant temozolomide after radiotherapy in newly diagnosed WHO grade 3 IDH-mutant astrocytoma.	Alliance for Clinical Trials in Oncology
Vorasidenib + temozolomide	NCT06478212	Phase Ib/II	Safety, tolerability, and preliminary efficacy of vorasidenib combined with temozolomide in recurrent grade 2–4 or newly diagnosed grade 4 IDH1/2-mutant glioma.	Institut de Recherches Internationales Servier
Vorasidenib + temozolomide	S095032-301	Phase III	Randomized, Double-blinded, Placebo-controlled, multicenter study of vorasidenib in combination with temozolomide (TMZ) in patients with Grade 4 IDH1- or IDH2-mutant astrocytoma. Radiographic progression-free survival, as assessed by a blinded independent review committee.	Institut de Recherches Internationales Servier
Vorasidenib + pembrolizumab	NCT05484622	Phase I	Safety lead-in and randomized perioperative study of vorasidenib combined with pembrolizumab in recurrent or progressive IDH1-mutant glioma.	Institut de Recherches Internationales Servier
ViCToRy	NCT05609994	Phase I	Vorasidenib in combination with a tumor-specific IDH1 peptide vaccine for recurrent IDH1-mutant lower-grade glioma.	Duke University
SIGMA	NCT05303519	Phase III	Efficacy and safety of safusidenib erbumine in participants with IDH1-mutant glioma.	Nuvation Bio Inc.Headquaters: New York, NY, United States
Olutasidenib + temozolomide	NCT06161974	Phase II	Olutasidenib with temozolomide as maintenance therapy in pediatric and young adult patients with newly diagnosed high-grade glioma, including diffuse intrinsic pontine glioma (DIPG), harboring IDH1 mutations.	Rigel Pharmaceuticals, Inc.: South San Francisco, CA, United States.

## Data Availability

No new data were created or analyzed in this study. Data sharing is not applicable to this article.
